# Diagnostic value of death certificates for case ascertainment of dementia: a population‐based assessment of mortality registry coding

**DOI:** 10.1002/alz70860_105491

**Published:** 2025-12-23

**Authors:** Frank J. Wolters, Peter P.M. Harteloh, Frank J.A. van Rooij, Pieter Bakx, Brenda C.T. Kieboom, Arfan Ikram

**Affiliations:** ^1^ Erasmus MC ‐ University Medical Center Rotterdam, Rotterdam, Zuid‐Holland, Netherlands; ^2^ Statistics Netherlands, The Hague, Zuid‐Holland, Netherlands; ^3^ Erasmus University Rotterdam, Rotterdam, Zuid‐Holland, Netherlands; ^4^ Erasmus University Medical Center, Rotterdam, South Holland, Netherlands

## Abstract

**Background:**

Ascertainment of dementia diagnosis in longitudinal studies is a strenuous and costly effort, which might be simplified using data from mortality registries. However, the diagnostic value of cause‐of‐death coding is uncertain, and could vary with patient characteristics and (co)morbidity.

**Method:**

We included 6280 participants of the population‐based Rotterdam Study, who were actively followed during life for the occurrence of dementia through repeated in‐person screening and extraction of medical records. For those who died between 1989‐2018 (mean age at death: 83.6 years, 57% women), we compared any diagnosis of dementia during life to their certified cause‐of‐death in the nationwide mortality registry in the Netherlands. We determined concordance of death certificates according to coding method (i.e., ICD‐9 and ICD‐10), place of death, and participant characteristics (age at death, dementia duration, and comorbidity). To validate observations, we surveyed 446 certifying physicians.

**Result:**

Between 1989‐2018, 1828 persons (29%) had a diagnosis of dementia during life. Specificity of death certificates for dementia diagnosis consistently exceeded 96%. Sensitivity was below 10% using ICD‐9, but increased to about 70% in more recent years using ICD‐10. Upon time‐series regression, improvements in diagnostic accuracy related significantly to introduction of ICD‐10 (odds ratio [95%CI]: 1.94 [1.08‐3.48]). Diagnostic accuracy varied substantially with age at death (OR per decade increase: 0.80 [0.66‐0.96]), dementia disease duration (OR [95%CI] for ≥6 years vs. 0‐2 years: 3.95 [2.56‐6.09]), and place of death. Moreover, comorbidity at time of death decreased the likelihood of accurately assessing dementia through causes of death (OR [95%CI] per additional illness: 0.82 [0.72‐0.93]), with accuracy reduced particularly in persons who had had stroke or heart failure. Dementia severity and duration were also reported by medical doctors as important factors weighing in on their decision to certify dementia as a cause of death.

**Conclusion:**

Death certificates have high specificity but limited sensitivity for diagnosis of dementia, in particular in older patients with recent dementia diagnosis or cardiovascular comorbidity. These findings aid to decide in longitudinal studies when to use cause‐of‐death statistics to obtain valid data on dementia diagnosis, and when in‐person screening or a combination of other data sources are required.

